# Circulating T Follicular Helper Cell Abnormalities Associated to Different Clinical Forms of Chronic Chagas Disease

**DOI:** 10.3389/fcimb.2020.00126

**Published:** 2020-03-31

**Authors:** Luz P. Quebrada Palacio, Esteban R. Fernández, Yolanda Hernández-Vásquez, Patricia B. Petray, Miriam Postan

**Affiliations:** ^1^Instituto Nacional de Parasitología “Dr. Mario Fatala Chabén”, ANLIS/Malbran, Buenos Aires, Argentina; ^2^Facultad de Medicina, IMPaM-Instituto de Investigaciones en Microbiología y Parasitología Médica (UBA-CONICET), Buenos Aires, Argentina; ^3^Consejo Nacional de Investigaciones Científicas y Tecnológicas (CONICET), Buenos Aires, Argentina

**Keywords:** T follicular helper cells, circulating Tfh subsets, CXCR3, CCR6, Human Chagas disease, *Trypanosoma cruzi*

## Abstract

Multiple perturbations of the immune response affecting a range of cells have been reported in *Trypanosoma cruzi*-infected individuals and associated to clinical manifestations of chronic Chagas disease. There is a paucity of knowledge about the role of T follicular helper (Tfh) cells in this infection. Here, we sought to characterize circulating Tfh (cTfh) cells in chronic Chagas disease patients and to identify potential associations with disease severity in humans. cTfh cells were characterized by flow cytometry in freshly isolated PBMCs from 7 *T. cruzi*-infected asymptomatic patients (ASYMP), 5 patients with chronic chagasic dilated cardiomyopathy (CCC) and 8 healthy controls, using antibodies against chemokine receptors CXCR5, CXCR3, CCR6, and CCR7. Our results showed significant expansion of CD4+CD45RO+CXCR5+CCR6+ cells in ASYMP and CCC patients, along with a contraction of CD4+CD45RO+CXCR5+CXCR3-CCR6- (cTfh2) cells. ASYMP patients further exhibited decreased CD4+CD45RO+CXCR5+CXCR3+CCR6- (cTfh1) cells and expanded CD4+CD45RO+CXCR5+CXCR3-CCR6+ (cTfh17) cells while CCC patients exhibited significantly increased frequencies of CD4+CD45RO+CXCR5+CCR7+ cells. Linear regression analysis revealed a positive trend of CD4+CD45RO+CXCR5+CXCR3+CCR6+ (cTfh1/17) cells and negative trends of cTfh1 and cTfh2 cells as disease was more severe. There was no correlation between the frequencies of cTfh cells and circulating CD19+IgD-IgG+ cells or serum levels of *T. cruzi*-specific IgG. These results demonstrate that the cTfh compartment of humans chronically infected with *T. cruzi* comprises expanded CCR6-expressing cells and reduced cTfh2 cells. The association of discrete phenotypic changes in cTfh subsets with different clinical forms suggests the potential contribution of T follicular helper cells to Chagas heart disease progression.

## Introduction

*Trypanosoma cruzi*, the etiological agent of Chagas disease, causes a broad spectrum of clinical outcomes, ranging from a mild, frequently asymptomatic acute illness with low mortality rate to a potentially life-threatening cardiomyopathy, digestive and/or neurological abnormalities in up to 30% of chronically infected individuals (WHO World Health Organization, 2018). Diverse parasite and host factors have been proposed to account for the differential clinical evolution of Chagas disease (Bryan et al., 2010; Dutra et al., 2014). *Trypanosoma cruzi* is a heterogeneous species and infection with different strains may result in different clinical outcomes (Quebrada Palacio et al., 2018).

Several parasite proteins have been shown to promote parasite invasion, replication and survival (Watanabe Costa et al., 2016). These molecules might also subvert the complement system and favor intracellular parasite growth, thus hampering the development of parasite-specific innate and adaptive immune responses (Cardoso et al., 2016). Several defects of the T cell compartment have been associated with the progression of asymptomatic *T. cruzi* infection to Chagas heart disease. Asymptomatic patients are known to develop balanced Th1/Th2/Th17/Treg adaptive immune responses, with effector cells producing proinflammatory cytokines such as IFN-γ, TNF-α and IL-17, and regulatory cells producing IL-10, while patients with the cardiac form of chronic Chagas disease exhibit a predominantly proinflammatory cytokine profile with limited production of IL-10 (Gomes et al., 2003; Vitelli-Avelar et al., 2008; Dutra et al., 2014). Various B cell defects have also been reported in *T. cruzi*-infected individuals, including polyclonal B cell activation, hypergammaglobulinemia and secretion of non-specific antibodies and autoantibodies, which have been suggested to delay parasite-specific humoral responses, thus contributing to parasite persistence and pathology (Minoprio et al., 1988; Bryan et al., 2010; Bermejo et al., 2011; Cunha-Neto et al., 2011).

CD4+ T follicular helper (Tfh) cells are a distinct subpopulation of cytokine-producing CD4+ T helper cells that express the chemokine receptor CXCR5 and provide support to cognate B cell responses in germinal centers of secondary lymphoid organs (Breitfeld et al., 2000; Schaerli et al., 2000; Nurieva et al., 2008). A population of CD4+CXCR5+ cells has also been described in the peripheral blood (cTfh) and proposed to represent the circulating counterpart of *bona fide* Tfh cells. As for Th1, Th2 and Th17 cells (Acosta-Rodriguez E et al., 2007), the chemokine receptors CXCR3 and CCR6 have been used to define functionally distinct cTfh cell subsets (Morita et al., 2011; Schmitt et al., 2014). In humans, CD4+CD45RO+CXCR5+CXCR3+CCR6- (cTfh1) cells are known to secrete mostly IFN-γ, while CD4+CD45RO+CXCR5+CXCR3-CCR6- (cTfh2) cells secrete IL-4, IL-5, IL-13 and IL-21, and CD4+CD45RO+CXCR5+CXCR3-CCR6+ (cTfh17) cells secrete mainly IL-17, IL-21, and IL-22. Dysregulation of the cTfh cell compartment has been reported in autoimmune diseases, cancer and infections associated with abnormal B cell responses (Boswell et al., 2014; Ueno et al., 2015). However, whether Tfh cells contribute to the generation of the abnormal B cell responses observed during *T. cruzi* infection remains to be elucidated (Minoprio et al., 1988; Fernández et al., 2014). The aim of this work was to provide an insight in the adaptive immune response elicited by *T. cruzi* in humans. To this end, various peripheral blood CD4+CD45RO+CXCR5+ cell subsets were assessed in adult patients with different clinical forms of chronic Chagas disease. The results showed expansion of CCR6+ cTfh cells and decreased cTfh2 cells in infected patients, regardless of the clinical status of the disease. Other phenotypic changes observed include expansion of cTfh17 cells and decreased cTfh1 cells in asymptomatic patients -but not in patients with chagasic dilated cardiomyopathy-, which suggest that dysregulation of Tfh cells might contribute to Chagas disease progression.

## Materials and Methods

### Study Population

A total of 31 adult participants were enrolled at the Clinical Facilities of Instituto Nacional de Parasitología “Dr. Mario Fatala Chabén,” Buenos Aires. The clinical status of enrolled individuals was determined by physical examination and clinical tests, including serologic testing for *T. cruzi* infection, electrocardiography, chest radiography, and echocardiography. Individuals who had positive serology for *T. cruzi* were grouped according to the classification of Kuschnir et al. (1985) into: (a) patients with no clinical manifestations of heart disease, a normal electrocardiogram and conserved cardiac silhouette in chest X-rays and echocardiogram (ASYMP; Kuschnir group 0; *n* = 12; mean age ± SD = 48.83 ± 12.37 years) and (b) patients with chronic Chagas dilated cardiomyopathy (CCC; Kuschnir group 2; *n* = 5; mean age ± SD = 48.4 ± 7.50); clinically healthy individuals with a negative test for *T. cruzi* infection (CTRL, *n* = 14; mean age ± SD = 41.71 ± 13.36) served as non-infected controls. Age of controls was matched as much as made possible to that of the infected individuals for each assay. A signed informed consent was obtained from all participants. All the research was conducted in accordance with the Declaration of Helsinki and the Council for International Organizations of Medical Sciences (CIOMS). The study protocol was approved by the Review Board of Instituto Nacional de Parasitología “Dr. Mario Fatala Chabén,” Administración Nacional de Laboratorios e Institutos de Salud Dr. Carlos G. Malbrán (IRB00006651).

### Isolation of PBMC and Flow Cytometry Assays

Freshly isolated peripheral blood mononuclear cells (PBMCs) were separated by density gradient centrifugation on Ficoll-Paque Plus (GE Healthcare, Uppsala, Sweden) and stained with mAbs against CD4-FITC (Biolegend), CD45RO-PerCp-Cy5.5 (Biolegend), CXCR5-APC (eBioscience), CCR7-PE-Cy7 (BD Pharmingen), PD1-Pac Blue (Biolegend), CXCR3-PE-Cy7 (Biolegend), CCR6-PE (Biolegend), CD19-PE-Cy7 (BD Pharmingen), IgD-PE (BD Pharmingen), and IgG-APC (BD Pharmingen), following the instructions of manufacturers. Cell viability was monitored in all samples by incorporating Fixable Viability Dye e-Fluor 780 (Invitrogen, Carlsbad, CA, USA) to the stains. At least 200,000 events per sample were collected at a FACSAria II flow cytometer (BD Immunocytometry Systems) and analyzed using FlowJo software (Tree Star, Inc., Ashland, OR, USA). Lymphocytes were identified by forward and side-angle light scatter characteristics. Non-specific fluorescence was defined with Fluorescence minus one (FMO) controls (Hulspas et al., 2009) (Supplementary Figure 1).

### Statistical Analysis

Statistical analysis was performed using GraphPad Prism (GraphPad Software, Inc., La Jolla, CA, USA). The Kolmogorov-Smirnov normality distribution test was applied to all data. ANOVA followed by Bonferroni or Kruskal Wallis followed by Dunns were used for multiple comparisons. Comparisons between groups were made using unpaired Student's *t*-test or Mann–Whitney test. Pearson or Spearman tests were used to determine correlation between study parameters. A simple linear regression test was used for trend analysis. Values of *p* < 0.05 were considered statistically significant.

## Results

### Identification of Circulating T Follicular Helper Cells in *Trypanosoma cruzi*-Infected Patients

**Figure 1:**
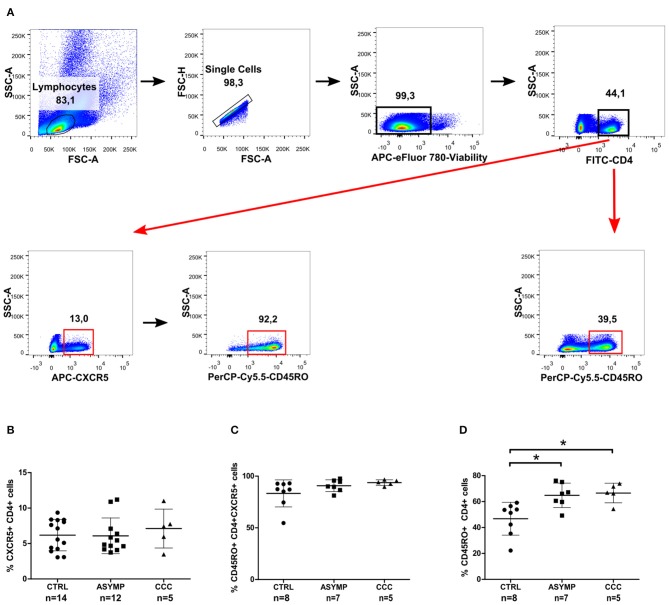
CXCR5 and CD45RO expression in peripheral blood CD4+ cells of chronic Chagas disease patients. Freshly isolated PBMCs were stained with fluorochrome-conjugated Abs against CD4, CXCR5 and CD45RO markers. **(A)** Gating strategy used for the identification of circulating CXCR5+ and CD45RO+ cells in CD4+ T cells. Data are presented as frequencies of CXCR5+ cells in CD4+ cells **(B)**, CD45RO+ cells in CXCR5+CD4+ cells **(C)** and CD45RO+ cells in CD4+ cells **(D)**. Each symbol represents an individual; mean values ±SD are represented by horizontal lines.*p < 0.05; ANOVA followed by Bonferroni.

**Figure 2:**
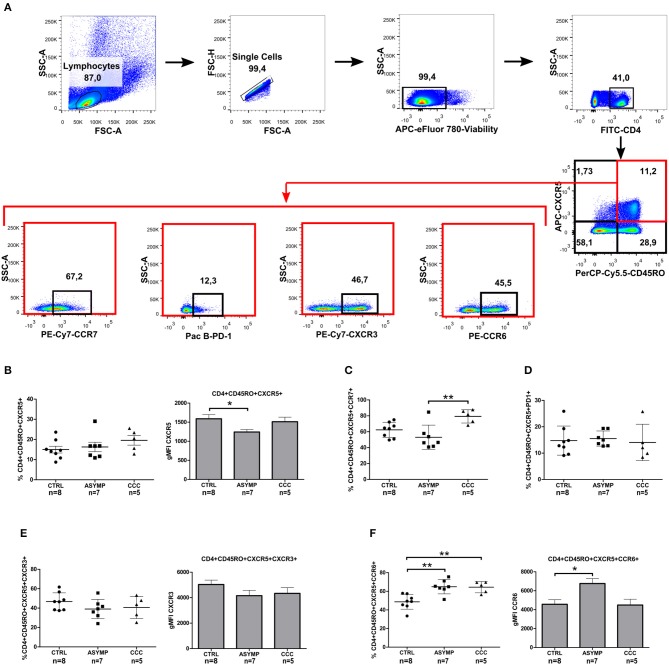
Phenotypic analysis of circulating memory T follicular helper cells in Chagas disease patients. **(A)** Gating strategy. CXCR5 and CD45RO double-positive CD4+ T cells were analyzed for the expression of CCR7, CXCR3, CCR6, and PD1 markers by flow cytometry. **(B)** Percentages of CD4+CD45RO+CXCR5+ cells and CXCR5 gMFI. **(C)** Percentages of CD4+CD45RO+CXCR5+CCR7+ cells. **(D)** Percentages of CD4+CD45RO+CXCR5+PD1+, **(E)** CD4+CD45RO+CXCR5+CXCR3+ and CXCR3 gMFI. **(F)** CD4+CD45RO+CXCR5+CCR6+ and CCR6 gMFI. Each symbol represents an individual; mean values ±SD are represented by horizontal lines. Bars represent the geometric mean fluorescence intensity of chemokine receptors CXCR5, CXCR3, and CCR6; error bars represent SD. *p < 0.05 and **p < 0.01; Kluskal–Wallis followed by Dunn's or ANOVA followed by Bonferroni.

It has been well established that CXCR5 expression is essential for migration of CD4+ T cells to B cell follicles, where they interact with cognate B cells and provide help for the generation of protective antibody responses (Breitfeld et al., 2000; Schaerli et al., 2000). We therefore asked whether chronic *T. cruzi* infection impacts on the circulating CD4+ cell population with homing capability to B cell follicles. To this end, we measured the expression of CXCR5 as frequency of overall circulating CD4+ cells in patients with different clinical forms of chronic Chagas disease and in healthy control subjects (Figure 1A). No statistically significant differences were found in the frequency of CXCR5+CD4+ cells among the study groups (Figure 1B). Since the CXCR5 marker has been shown to be expressed by recently activated naïve CD4+ cells and a subset of long-lived memory CD4+ cells (Schaerli et al., 2001; Nurieva et al., 2008; Schmitt et al., 2014), we next measured the expression of CD45RO on CXCR5+CD4+ cells. Most circulating CXCR5+CD4+ cells co-expressed the memory cell marker in our study population (Figure 1C), as described by other authors (Breitfeld et al., 2000; Schaerli et al., 2000; Schmitt et al., 2014). Further analysis of CD45RO expression on the overall circulating CD4+ cell population showed a significantly higher frequency of CD4+CD45RO+ cells in *T. cruzi*-infected subjects, as compared to control subjects, irrespective of the clinical status of Chagas heart disease (*P* = 0.0041; Figure 1D). Thus, cTfh cells were defined by the co-expression of CXCR5 and CD45RO within the CD4+ cell compartment (Figure 2A). The frequencies of CD4+CD45RO+CXCR5+ cells were not different among the study groups. However, the CXCR5 gMFI in ASYMP patients was significantly lower as compared to control subjects (*P* = 0.0438; Figure 2B).

To determine whether *T. cruzi* infection leads to a change in the phenotype of cTfh cells, we next examined the expression of CCR7 (the chemokine receptor that controls homing to secondary lymphoid organs) and PD-1 (a molecule highly expressed by activated T cells) (Sallusto et al., 1999; Keir et al., 2008). CCC patients exhibited a statistically significant higher frequency of circulating CD4+CD45RO+CXCR5+CCR7+ cells, as compared to ASYMP patients (*P* = 0.0048; Figure 2C). On the other hand, most circulating CD4+CD45RO+CXCR5+ cells did not express PD-1, indicating that cTfh cells were in a resting status, irrespective of the infection and clinical condition (Figure 2D). Afterward, we evaluated the expression of chemokine receptors CXCR3 and CCR6 (classically linked to the production of IFN-γ and IL-17, respectively; Singh et al., 2008; Groom and Luster, 2011) on CD4+CD45RO+CXCR5+ cells. No statistically significant differences were found in the frequency of CD4+CD45RO+CXCR5+CXCR3+ cells and CXCR3 gMFI levels among the study groups (Figure 2E). However, the frequencies of CD4+CD45RO+CXCR5+CCR6+ cells were significantly higher in both patient groups, as compared to controls (*P* = 0.0008; Figure 2F). Besides, CCR6 gMFI on CD4+CD45RO+CXCR5+CCR6+ cells in ASYMP patients was significantly higher than that in control subjects (*P* = 0.0121). Noteworthy, the proportions of CD4+CD45RO+CXCR5+CCR6+ cells in ASYMP and CCC patients were significantly higher than those of CD4+CD45RO+CXCR5+CXCR3+ cells (*P* = 0.0001 and *P* = 0.0033, respectively; Student's *t*-test), as opposed to the balanced distribution of these cells observed in healthy controls (*P* = 0.6550; Student's *t*-test). Altogether, these data suggest that the infection with *T. cruzi* induces a shift of the cTfh compartment toward a Th17-like phenotype.

### Chronic Chagas Disease Is Associated With a Defective cTfh Cell Subset Composition

**Figure 3:**
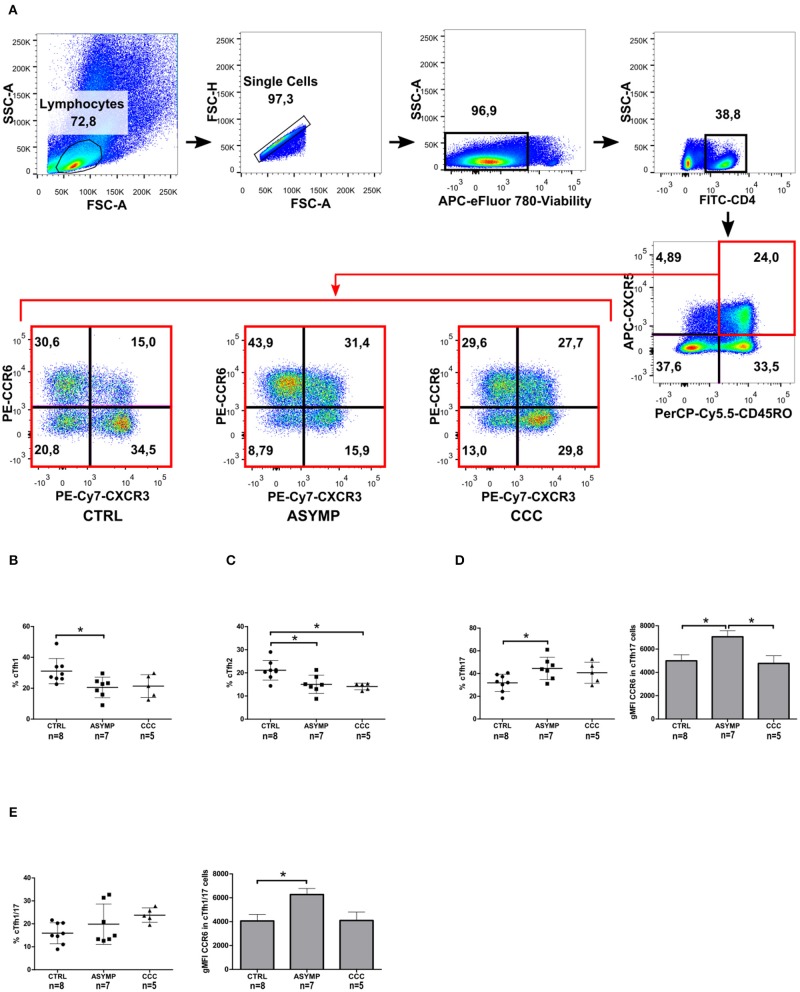
Altered distribution of circulating T follicular helper subsets during chronic *T. cruzi* infection. **(A)** Gating strategy. cTfh memory cells were analyzed for the differential co-expression of CXCR3 and CCR6 by flow cytometry. Frequencies of cTfh1 **(B)**, cTfh2 **(C)**, cTfh17 **(D)** and cTfh1/17 cells **(E)**. Each symbol represents an individual; mean values ±SD are represented by horizontal lines. Bars represent the CCR6 gMFI; error bars represent SD. *p < 0.05; ANOVA followed by Bonferroni.

The differential co-expression of CXCR3 and CCR6 was then used to define functionally distinct cTfh subsets (Figure 3A). ASYMP patients showed decreased cTfh1 cell frequencies, as compared to controls (*P* = 0.0282; Figure 3B); nevertheless, linear regression analysis revealed a negative trend in the frequency of these cells as the disease severity increased (*P* = 0.0271, slope = −3.747). No differences in CXCR3 gMFI levels were observed among the study groups. The frequency of cTfh2 cells in ASYMP and CCC patients was significantly lower, as compared to controls (*P* = 0.0043; Figure 3C), with a negative trend in these cells as the infection was more severe (*P* = 0.0026, slope= −3.747). Conversely, the frequency of cTfh17 cells was significantly increased in ASYMP patients, as compared to controls (*P* = 0.0359; Figure 3D). Additionally, CCR6 gMFI levels on these cells were significantly higher in ASYMP patients, as compared to both CCC patients and controls (*P* = 0.0178). The frequency of cTfh1/17 (Liu et al., 2017) cells was similar among the study groups (Figure 3E), though a positive trend was observed with disease progression (*P* = 0.0327, slope 3.921). In these cells, CXCR3 gMFI levels were similar among the study groups; however, CCR6 gMFI levels were significantly higher in ASYMP -but not in CCC- patients, as compared to controls (*P* = 0.0182; Figure 3E).

**Figure 4:**
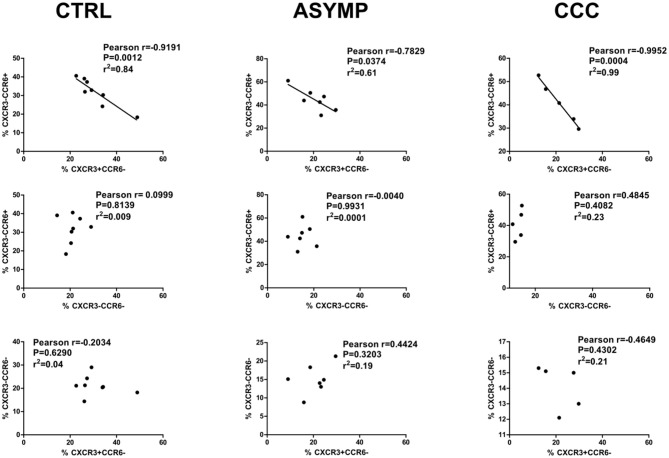
Correlation analysis between cTfh subset frequencies. cTfh17 cell frequencies inversely correlate with cTfh1 cell frequencies in Chagas disease patients and healthy subjects. There is no correlation of cTfh17 vs. cTfh2 cell frequencies, and cTfh1 vs. cTfh2 cell frequencies. *p* < 0.05 was consider significant; Pearson correlation coefficient test.

Previous studies from our lab have shown a progressive decrease in *T. cruzi*-specific CD4+ IFN-γ+ cell numbers as Chagas heart disease was more severe (Albareda et al., 2009). Other authors have reported that the Th1 effector cytokine IFN-γ inhibits the development of Th17 effector responses (Harrington et al., 2005). T-bet, the transcription factor involved in the differentiation of Th1 cells has been demonstrated to inhibit *T. cruzi*-specific CD4+ Th17 cell responses *in vivo* (Guo et al., 2009). We here analyzed the relationship between different cTfh subsets by linear regression and found that the frequency of cTfh1 cells inversely correlated with that of cTfh17 cells in infected patients as well as in controls (Figure 4). No correlation between cTfh1 vs. cTfh2, and cTfh2 vs. cTfh17 cell frequencies was found. Similarly, the frequency of CD4+CD45RO+CXCR5+ cells did not correlate with the frequency of circulating CD19+IgD-IgG+ B cells (Supplementary Table 1). We also failed to detect a relationship between the frequencies of cTfh subsets and serum levels of *T. cruzi*-specific IgG antibodies, as measured by ELISA (data not shown).

As IFN-γ and IL-17 were proposed to play critical roles in the pathogenesis of Chagas disease cardiomyopathy (Magalhães et al., 2013; Dutra et al., 2014), we extended our phenotypic analysis of CXCR3 and CCR6 expression to CD4+, CD4+CD45RO+ and CD4+CD45RO+CXCR5- (non-follicular memory) cells. No differences were observed in the frequencies of CD4+ cells and CD4+CD45RO+ cells expressing either marker among the study groups (Supplementary Table 1). The proportion of CXCR3+ cells within the CD4+CD45RO+CXCR5- compartment was significantly higher than that of CCR6+ cells in healthy controls (*P* = 0.0012; Student *t*-test), but not in infected patients (*p* > 0.05). These data suggest that Th1 polarization of non-follicular memory CD4+ cells does not occur in chronic Chagas disease patients. We next measured CXCR3 and CCR6 co-expression in CD4+CD45RO+CXCR5- cells and found similar frequencies of CXCR3+CCR6-, CXCR3-CCR6-, CXCR3-CCR6+ and CXCR3+CCR6+ cells among the study groups (Supplementary Table 1).

## Discussion

The relevance of abnormal T and B cell responses in the progression of chronic Chagas disease has received considerable attention. Multiple T cell subsets and cytokines seem to be involved in the pathogenesis of Chagas cardiomyopathy, but the exact mechanisms leading to parasite persistence and tissue damage have not been fully established. Classically, effector T helper cells include distinct subsets which can be distinguished by specific activating transcription factors, the profile of cytokines they secrete upon re-stimulation and the chemokines defining their migration properties (Deenick et al., 2011). T follicular helper cells represent a particular lineage of CD4+ helper cells that provide essential support to cognate B cells for proliferation, somatic hypermutation, class-switch recombination, and selection of high affinity B cells in germinal centers of secondary lymphoid organs (Crotty, 2015). In this study, we evaluated the expression of the chemokine receptors CXCR3 and CCR6 on CD4+CD45RO+CXCR5+ cells from patients chronically infected with *T. cruzi*. The results of our approach exposed a dysbalanced cTfh cell composition, which includes a progressive contraction of cTfh1 and cTfh2 cells, expansion of cTfh1/17 cells and increased frequency of CCR6+ cells as disease was more severe. Further analysis of cTfh17 and cTfh1/17 exposed the association of increased CCR6 cell expression with asymptomatic disease, supporting the concept that IL-17 plays a protective role against the progression to Chagas cardiomyopathy proposed by other authors (Magalhães et al., 2013). The contraction of cTfh1 cells was more marked in asymptomatic patients, and it was accompanied by the expansion of cTfh17 cells. It is plausible that the shift toward a Th17-like phenotype observed occurs during early stages of CD4+ T cell differentiation, since IFN-γ and IL-4 are known to antagonize naive, but not mature Th17 cells (Harrington et al., 2005). The exact mechanisms involved in the generation of expanded cTfh17 cells and contracted cTfh1 and cTfh2 cells during chronic *T. cruzi* infection remains to be determined. This is the first report of the impact of chronic *T. cruzi* infection on the population of circulating Tfh cells. Although yet to be established, the altered composition of cTfh cells may reflect changes in germinal center dynamics that could play a role in the pathogenesis of Chagas disease. A better understanding of the functionality of these cells will likely provide new and important clues about the immune response against *T. cruzi* and the potential implication of cTfh cells in the progression to Chagas disease cardiomyopathy.

The differentiation of activated B cells into immunoglobulin-secreting cells in tonsils was demonstrated to be largely mediated by Tfh cell-derived IL-21 (Bryant et al., 2007). Different authors have demonstrated that peripheral blood Tfh17 and Tfh2 cells produce IL-21 more efficiently than other circulating Tfh and Th subpopulations, and are thus considered efficient B helper cells (Morita et al., 2011; Schmitt et al., 2014). In contrast with reports on humoral responses in autoimmune diseases (Morita et al., 2011), in this work we failed to demonstrate any association of cTfh subsets with the frequency of IgG+ mature B cells *ex-vivo* and the levels of serum *T. cruzi*-specific antibodies. This phenomenon could be attributed to humoral *T. cruzi*-specific responses being regulated by mechanisms other than Tfh cells, including T cell-independent pathways, as proposed for human HIV infection (Boswell et al., 2014). Interestingly, massive extrafollicular B cell responses were described as an important source of non-specific antibodies in mice acutely infected with *T. cruzi* (Bermejo et al., 2011). Otherwise, peripheral blood Tfh cells in chronic *T. cruzi* infection might not fully represent the pool of Tfh cells residing in germinal centers.

Naïve CD4+ T cells are known to constitutively express CCR7, a key molecule for cell migration toward CCL19 and CCL21 ligands expressed in the T-zones of secondary lymphoid organs. Diverse cytokines and inflammatory mediators have been shown to modulate the expression of CCR7 and immune cell activation (Comerford et al., 2013). The attenuation of CCR7 expression and the induction of CXCR5 facilitate the entry of CD4+ cells into B cell follicles and GC in response to CXCL13 expression; CCR7 recycles back to the cell membrane after egressing from lymph nodes in a sphingosine-1-phosphate receptor-1 (S1P1)- dependent manner (Matloubian et al., 2004). Conversely, the maintenance of CCR7 expression was shown to inhibit the access of CXCR5+ cells to follicles (Haynes et al., 2007). Chronic chagasic cardiomyopathy is characterized by myocardial cell necrosis, fibrosis and active inflammatory process involving CD8+ and CD4+ T cells, NK cells and macrophages which secrete mainly proinflamatory cytokines (Dutra et al., 2014). It is possible that inflammatory mediators associated with cardiac lesions induce the expression of CCR7 on CXCR5+ cells, promoting the re-circulation of CCR7+ cTfh cells and their accumulation in the peripheral blood.

An additional finding of our study was the expansion of peripheral blood CD4+CD45RO+ cells associated with chronic *T. cruzi* infection, which contrasts with the unaltered proportion of CD4+CD45RO+ cells *ex-vivo* reported by Fiuza et al. (2009). It is possible that this discrepancy be due to the influence of parasite and host genetic diversity on the outcome of human Chagas disease, as suggested by other authors (Santos D et al., 2009; Zingales et al., 2012; Poveda et al., 2014).

In summary, in this work we characterized circulating Tfh cells in patients chronically infected with *T. cruzi* and demonstrated an imbalance in the distribution of cTfh subsets associated with the clinical forms of Chagas disease. The major weakness of the present study is the relatively low number of patients with dilated chagasic cardiomyopathy included in the analysis. Moreover, we were unable to obtain data on the cytokine profile and functionality of cTfh cells that could lead to a better understanding of their role in the pathogenesis of chronic Chagas heart disease. However, our detailed phenotypic characterization provides indirect evidence of a potential role of Tfh cells in the abnormal immune responses against *T. cruzi* (Fernández et al., 2014), establishing the basis to perform further studies to assess these processes.

## Data Availability Statement

The datasets generated for this study are available on request to the corresponding author.

## Ethics Statement

The studies involving human participants were reviewed and approved by the Review Board of Instituto Nacional de Parasitología Dr. Mario Fatala Chabén, Administración Nacional de Laboratorios e Institutos de Salud Dr. Carlos G. Malbrán (IRB00006651). The patients/participants provided their written informed consent to participate in this study.

## Author Contributions

MP conceived the project and designed research. MP and LQ performed the experiments, interpreted data, and wrote the paper. YH-V enrolled and performed clinical evaluation of participants. EF, PP, and YH-V interpreted data, validated, and edited the paper. MP and PP acquired funding.

### Conflict of Interest

The authors declare that the research was conducted in the absence of any commercial or financial relationships that could be construed as a potential conflict of interest.
