# Supplementary material for: Circulating T Follicular Helper Cell Abnormalities Associated to Different Clinical Forms of Chronic Chagas Disease
Source: Front Cell Infect Microbiol. 2020 Mar 31;10:126. doi: 10.3389/fcimb.2020.00126 (PMC7136390; doi:10.3389/fcimb.2020.00126)
Supplement: Supplementary file 1 [file Data_Sheet_1.pdf]

Supplementary Table 1. CXCR3 and CCR6 expression in non-follicular T helper cells from *T.cruzi* chronically infected patients and controls. Freshly isolated PBMCs were stained with fluorochrome-conjugated Abs to CD4, CD45RO, CXCR5, CXCR3 and CCR6. Data represent mean±SD of frequencies for each phenotype. [£]p<0.05; ANOVA followed by Bonferroni. [§] P=0.0012 vs. CD4+CD45RO+CXCR5-CCR6+; t test.

**Phenotype**	**CTRL**	**ASYMP**	**CCC**	**P[£]**
CD4+CXCR3+	32.98±10.6	39.77±11.4	41.18±8.51	0.2017
CD4+CCR6+	25.79±10.1	32.55±13.6	32.38±4.51	0.2822
CD4+CD45RO+CXCR3+	58.07±8.10	50.72±13.5	53.08±13.9	0.3645
CD4+CD45RO+ CCR6+	48.17±6.05	43.68±15.4	46.36±4.71	0.7009
CD4+CD45RO+CXCR5-CXCR3+	63.26±7.35 [§]	52.75±12.4	55.22±15.1	0.2103
CD4+CD45RO+CXCR5-CCR6+	46.55±9.14	37.3±16.7	42.04±4.14	0.3351
CD4+CD45RO+CXCR5-CXCR3+ CCR6-	35.17±8.18	33.37±13.4	32.38±13.1	0.7253
CD4+CD45RO+CXCR5-CXCR3- CCR6-	18.52±5.65	28.48±11.4	26.36±11.0	0.0567
CD4+CD45RO+CXCR5-CXCR3- CCR6+	18.06±4.81	16.22±6.28	18.70±6.80	0.7405
CD4+CD45RO+CXCR5-CXCR3+CCR6+	26.51±5.86	21.90±10.42	22.56±5.01	0.4728
CD19+IgD-IgG+	10.35±6.45	11.28±4.44	8.59±2.35	0.4804

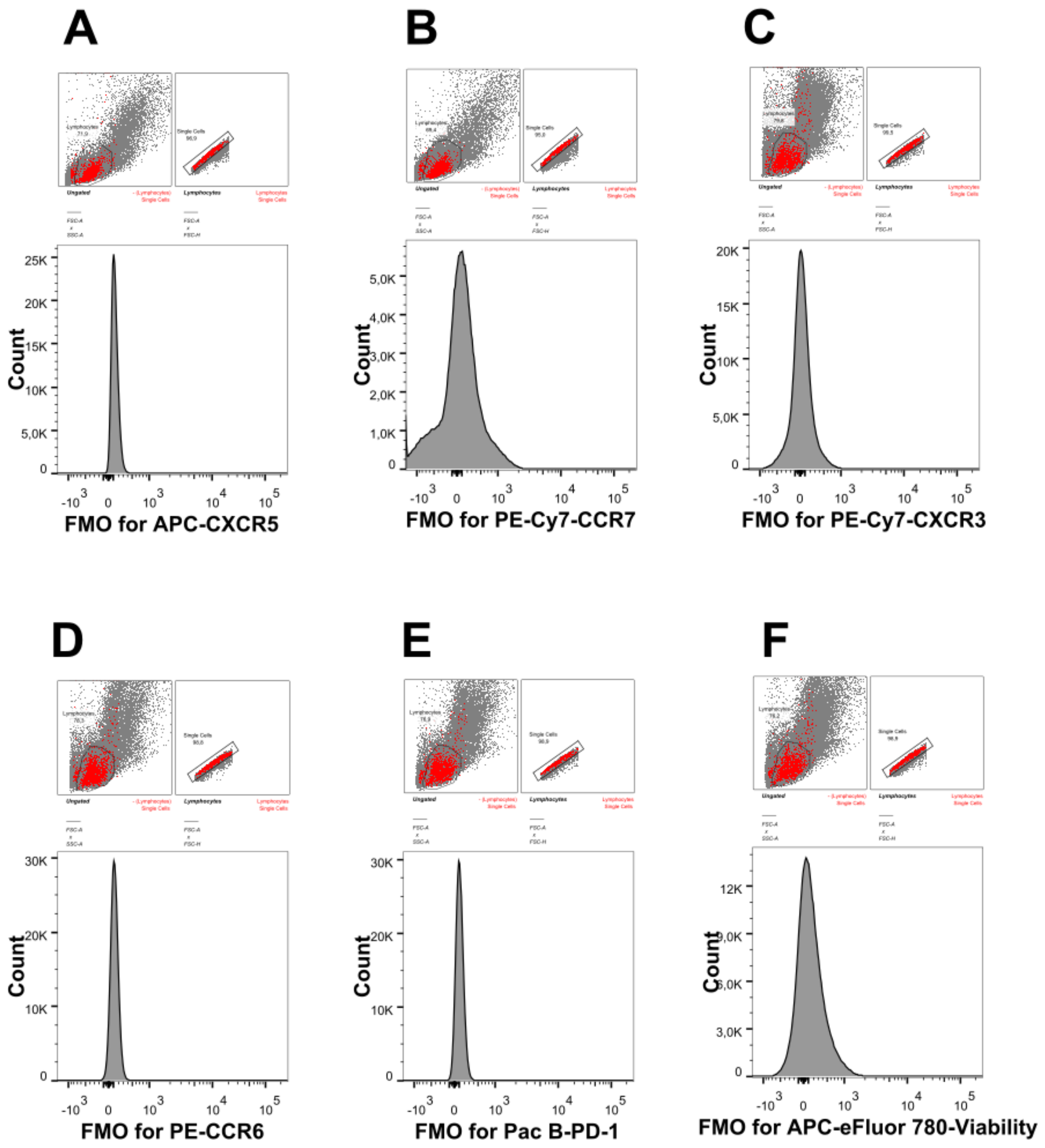

Supplementary Figure 1. Identification of non-specific fluorescence by Fluorescence Minus One (FMO) controls. Samples were stained with all fluorescent markers with the exception of CXCR5-APC (A), CCR7-PE-Cy7 (B), CXCR3-PECy7 (C), CCR6-PE (D), PD-1-PacBlue (E) or eFluor 780-viability- APC (F).
